# Mental Health Interventions for Young People Living With HIV/AIDS in Sub-Saharan Africa: A Systematic Review

**DOI:** 10.1155/arat/5323539

**Published:** 2025-05-01

**Authors:** Samuel Adjorlolo, Dorothy Serwaa Boakye, Eugenia Xatse, Vincent Valentine Akorli, Paul Kwame Adjorlolo, Yvonne Yawa Battanis, Abigail Bempomaa Frempong, Lydia Kaki Ocansey, Cecilia Yeboah

**Affiliations:** ^1^Department of Mental Health Nursing, College of Health Sciences, University of Ghana, Legon, Greater Accra Region, Ghana; ^2^Research and Grant Institute of Ghana, Legon, Greater Accra Region, Ghana; ^3^Department of Health Administration and Education, University of Education, Winneba, Central Region, Ghana; ^4^Department of Nursing, Knutsford University College, Accra, Greater Accra Region, Ghana; ^5^Department of Biostatistics, School of Public Health, University of Ghana, Legon, Greater Accra Region, Ghana; ^6^College of Community Health Nursing, Winneba, Central Region, Ghana; ^7^Nursing and Midwifery Training College, Koforidua, Eastern Region, Ghana

**Keywords:** adolescents, Africa, HIV, interventions, mental health, systematic review, youth

## Abstract

**Introduction:** Young people (aged 15–24) living with HIV/AIDS (YPLHIV) in sub-Saharan Africa (SSA) experience higher rates of mental health conditions compared to their uninfected peers. Research and practitioners have expressed interest in designing and implementing mental health interventions to improve the mental health and well-being of this vulnerable population. However, there is limited effort to systematically synthesize existing evidence on mental health interventions for YPLHIV to address salient questions relating to effectiveness, characteristics, practice issues among others to inform practice, and future research endeavors. This systematic review was conducted to take stock and synthesize existing data to address the above issues.

**Methods:** This review was conducted per the Preferred Reporting Items for Systematic Reviews and Meta-Analyses (PRISMA) guidelines. A comprehensive search strategy was implemented, utilizing five electronic databases and gray literature repositories. Studies (1) from SSA that focused on young adults with HIV/AIDS and (2) examined the effectiveness of interventions designed to enhance mental health outcomes and treatment adherence were included. Two independent reviewers were involved in the study selection, data extraction, and quality assessment, resolving discrepancies by consensus or consultation. Data were presented using narrative syntheses.

**Results:** Eight studies met the inclusion criteria, with a total sample size of 1510 participants, reporting on interventions from six African countries. The interventions were categorized as follows: cognitive behavioral therapy–based, family-based, peer support, and community-based. The interventions showed mixed effectiveness for depression, with three studies demonstrating significant improvements while four showed no substantial change. The only study on improving anxiety reported promising results. Four interventions positively influenced ART adherence, although with varying magnitudes. Peer support, family-based approach, and digital (mobile phone) approaches were identified as effective strategies.

**Conclusions:** The review showed promising approaches to improve mental health in YPLHIV, namely, through peer support, family-based, and digital (i.e., mobile phone) approaches. Although the results were mixed for depression and limited but positive for anxiety, several interventions improved ART adherence. However, the modest evidence base and varied measurement approaches necessitate more research across SSA.

## 1. Background

Globally, 38.4 million people are living with HIV, of which in 2021 approximately 4.8 million were young people aged 10–24 years [1, 2]. Sub-Saharan Africa (SSA) contributes substantially to the HIV burden, reflected in the high HIV prevalence (e.g., over 80%), mortality, and morbidity [3]. The diagnosis of HIV among young people potentiates a cascade of mental health, behavioral, and cognitive problems that impact all aspects of HIV prevention, treatment, and management [4]. A recent systematic review in SSA found that 25% of young people living with HIV/AIDS (YPLHIV) screened positive for any psychiatric disorder, whereas 30%–50% presented with emotional and behavioral difficulties or significant psychological distress [5]. This finding compares to a recent global meta-analytic study prevalence of 26.07% [6]. The burden of mental health problems differs greatly across the countries in SSA, with about 27% and 12% of adolescents living with HIV (ALHIV) in South Africa endorsing elevated symptoms of depression and anxiety, respectively [7], whereas 15% of HIV-positive adolescents in Botswana reported suicidal ideation, with female adolescents endorsing more thoughts of suicidality/self-harm [8]. Similarly, the prevalence of depressive symptoms among young people in Ethiopia was estimated at 35.5% [9], while in Uganda, depressive and anxiety symptoms were reported by 19.8% and 8.9% of YPLHIV, respectively [10].

Mental health problems in HIV/AIDS (1) contribute significantly to poor health outcomes by negatively impacting adherence to treatment, (2) negate the adoption of safe sexual practices and behaviors, and (3) hinder school attendance and academic achievement among young people [11]. These observations have potentiated discussions and facilitated calls for evidence-based intervention programs to improve mental health and related outcomes for young people in SSA [12]. Historically, mental health research, infrastructure, and service delivery are underdeveloped and poorly organized in SSA due to a confluence of factors, including low political will, overconcentration on other disease conditions (e.g., infectious disease), and low budgetary allocation. However, the last 2 decades have witnessed a growing trend in mental health publications and initiatives on the continent, including mental health interventions targeting HIV/AIDS [13, 14]. These interventions range from psychosocial support programs to evidence-based psychotherapies, such as cognitive-behavioral therapy (CBT) and interpersonal therapy (IPT) [15]. As a burgeoning area in HIV research in SSA, it is extremely important to take stock and characterize the literature to improve our understanding of existing programs, including their effectiveness as well as advance future discourse. Previous systematic reviews on adolescents and YPLHIV (ALHIV) have predominantly occurred within global or low- and middle-income country contexts [16–18], with limited focus specifically on SSA. While related reviews have explored mental health interventions for adolescents in the region, such as the review by Mabrouk et al. [19], which examined mental health interventions for adolescents in SSA more broadly, our review offers a unique contribution by focusing specifically on YPLHIV, providing a comprehensive analysis of mental health interventions tailored to this specific population and addressing the critical gap in understanding contextually adapted interventions for ALHIV in SSA. Consequently, significant knowledge gaps remain regarding the types of mental health interventions being implemented for ALHIV, their specific characteristics, and how they are developed or adapted for local SSA contexts. Our systematic review aimed to address these critical gaps by offering an in-depth examination of existing mental health interventions specifically designed for YPLHIV in the SSA region. It follows that a systematic synthesis of existing interventions would provide valuable insights to inform future programming and research in this field.

## 2. Methods

### 2.1. Design

The systematic review adhered to the revised 2020 edition of the Preferred Reporting Items for Systematic Reviews and Meta-Analysis (PRISMA) standards [20]. We defined and refined our review question and inclusion/exclusion criteria based on the Cochrane Handbook for Systematic Reviews of Interventions. A modified Population, Intervention, Comparison, and Outcome (PICO) framework, developed by the Cochrane Collaboration guidelines, was used to structure the key concepts as follows: *P* is YPLHIV in SSA; *I* is mental health interventions; *C* is YPLHIV not receiving mental health interventions; and *O* refers to primary outcomes (i.e., depression and anxiety) and secondary outcomes (adherence to ART). This review is registered with PROSPERO (CRD42024538957). The review protocol has been published elsewhere (XXXX).

### 2.2. Eligibility Criteria

The eligibility criteria were guided by the PICO framework and the Cochrane Handbook Collaboration guidelines. Additionally, the review considered criteria such as study types, settings/contexts, timelines, and language restrictions.

### 2.3. Inclusion Criteria

#### 2.3.1. Population

Studies involving young people aged 10–25 years living with HIV/AIDS. Given the scarcity of studies on this demographic, we included 2 studies that combined children and adolescents aged 8–18 years, as they met all other inclusion criteria.

#### 2.3.2. Intervention

We included studies that evaluated psychosocial or mental health interventions aimed at improving mental health outcomes (depression, anxiety, and suicide) and adherence to ART among YPLHIV.

#### 2.3.3. Comparison

Studies with or without comparator groups were eligible for inclusion in the systematic review.

#### 2.3.4. Outcome

Studies that focused on the impact of mental health interventions on depression, anxiety, and suicidal behaviors (primary outcomes) and adherence to ART (secondary outcome) were included.

#### 2.3.5. Study Design

Randomized controlled trials, quasiexperimental studies, pilot interventions, and prepost studies evaluating the effectiveness, acceptability, and/or feasibility of mental health interventions were considered for inclusion in the review.

#### 2.3.6. Setting/Context

Only studies conducted in SSA countries were included in the review.

#### 2.3.7. Language

Studies published in English (due to the cost and constraints of translation) were included.

#### 2.3.8. Timeframe

Studies published from the year 2004 to June 2024 were included, given the focus on providing insights into recent developments in mental health intervention programming for YPLHIV.

### 2.4. Exclusion Criteria

• Studies that focused solely on biomedical or pharmacological interventions without including a psychosocial or mental health component were excluded.
• Review articles, editorials, commentaries, case reports, and qualitative studies that did not evaluate an intervention were also excluded.
• Any studies where the full text was not available or accessible, either through online databases or other means, were not included in the analysis.

### 2.5. Search Strategy and Electronic Database Search

For this review, we determined the PICO components and cross-referenced them with search terms from comparable prior reviews to ensure thoroughness. Composite search terms were created, incorporating controlled vocabulary specific to the selected databases (e.g., MeSH terms for PubMed) along with keywords related to the population (e.g., Adolescen^∗^ OR “young adult^∗^” OR teen^∗^ OR youth OR “young people” AND HIV OR HIV/AIDS OR “HIV infected”), types of interventions (e.g., “Mental health intervention^∗^” OR “cognitive behavio? ral therap^∗^” OR “psychosocial intervention^∗^” OR “psychological intervention^∗^”), and context (“sub Saharan Africa” OR Africa). After testing the search strategy in three databases (PubMed, Scopus, and MEDLINE), we convened as a group and, following further discussions, decided that modifications were necessary to limit unrestricted access to articles. This was accomplished by adjusting the PICO framework to focus on Population, Intervention, and Settings. The outcomes were assessed during the screening and inclusion of articles. This indicates that the final search strategy employed across the selected databases was adjusted to exclude keywords associated with the primary and secondary outcomes (see Table 1).

The researchers (S.A. and D.S.B.) conducted a comprehensive literature search across five prominent electronic databases recommended by Cochrane. We developed a customized search strategy to identify relevant studies for inclusion in the review. These databases included PubMed, Scopus, MEDLINE, CINAHL, and Web of Science. Additional searches were conducted in African Journals Online, Google Scholar, library catalogs, conference proceedings, and clinical trials databases such as ClinicalTrials.gov, dissertation abstracts, and institutional databases such as UNAIDS and World Health Organization (WHO) to minimize publication bias. Relevant journals and reference lists of included studies were also hand-searched. Searches on the databases were limited to articles published from January 1, 2003, to June 3, 2024. Additional searches were also completed on June 15, 2024. The search was restricted to the last 20 years to understand recent developments in mental health intervention programming for YPLHIV.

### 2.6. Data Management and Screening

**Figure 1:**
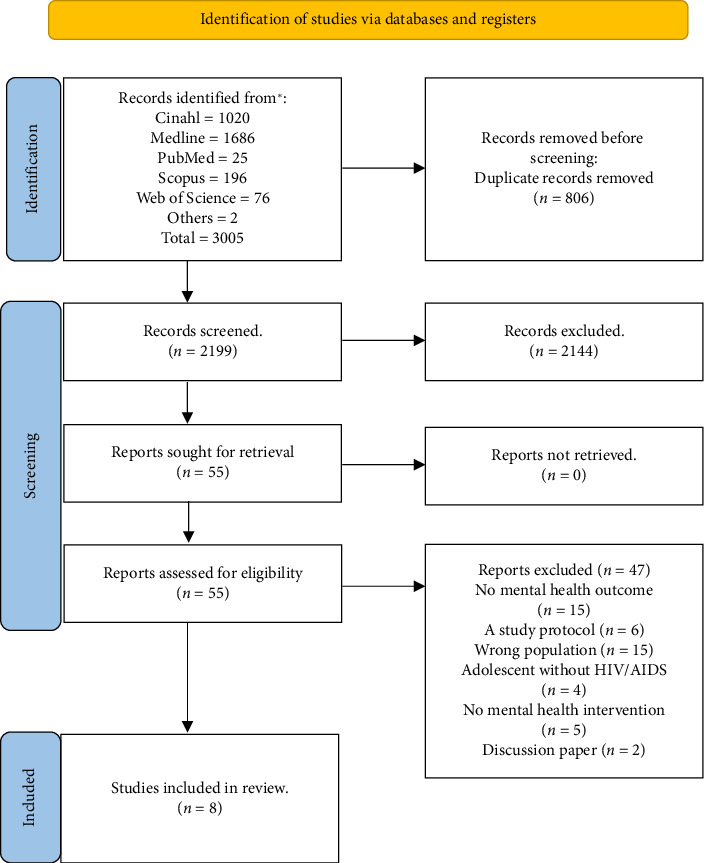
Schematic representation of systematic review of research on mental health interventions for adolescents living with HIV/AIDS in sub-Saharan Africa.

The search results were imported into Rayyan, a reference management software, where duplicate records were identified and removed. Two independent reviewers (S.A. and E.X.) screened the titles and abstracts against the inclusion and exclusion criteria, resolving any disagreements through discussion or consultation with a third reviewer. Full-text articles of potentially eligible studies were retrieved and assessed for inclusion by the two independent reviewers, with the third reviewer (D.S.B.) involved in case of disagreements. Reasons for study exclusion at this screening phase were documented and detailed in a PRISMA flow diagram (see Figure 1).

### 2.7. Data Extraction

A standardized data extraction form (Excel spreadsheet), adapted from previous systematic reviews [17, 18], was used to record relevant information from the included studies. Two reviewers' (D.S.B. and E.X.) pilot-tested the data extraction format, discussed, and iteratively refined it. Data extraction was conducted independently by two reviewers (D.S.B. and E.X.), with discrepancies resolved through discussion and a third reviewer's involvement. Extracted data included study characteristics (e.g., author, year, country, and study design), population details, intervention details, key findings on intervention effectiveness, and implementation approaches (Tables 2 and 3).

### 2.8. Data Synthesis

A qualitative content analysis approach [21] was used to collate the extracted data, categorizing and comparing similarities, differences, and relationships regarding mental health interventions and their impact on mental health outcomes and ART adherence across different studies. The data were synthesized by identifying themes and concepts relevant to the review question. To address intervention heterogeneity, we categorized interventions based on their primary therapeutic approach and analyzed outcomes separately for different mental health conditions. Interventions addressing faulty or maladaptive cognition and behaviors were categorized as cognitive behavior–based approach, whereas those focusing on improving family were grouped as family-based therapy. The duration in implementing the intervention was documented and considered in the analysis of effectiveness.

### 2.9. Risk of Bias Assessment

Eligible studies were assessed for quality using standardized criteria suitable for their respective study designs. Two reviewers (S.A. and D.S.B.) independently evaluated each study's methodological quality and resolved any discrepancies through discussion. If needed, a third reviewer (P.K.A. or V.V.A.) provided the final judgment on the study's rating. Randomized controlled trials were evaluated with the Cochrane Risk of Bias tool, which checks for potential biases such as randomization, allocation concealment, and selective reporting [22]. Nonrandomized studies were assessed using a modified version of the Newcastle–Ottawa Scale [23]. When study reports lacked sufficient information, the judgment “UNCLEAR” was given to the specific risk of bias items. Each study was then assigned a final quality rating of “low,” “moderate,” or “high” based on the comprehensive evaluation of bias risk. The Cochrane Risk of Bias tool is a 6-item checklist with an overall score of 6. Scores of 0–2, 3–4, and 5–6 were rated as low, moderate, and high quality, respectively. The Newcastle–Ottawa Scale is a 9-item scale with scores of 0–3, 4–6, and 7–9 indicating low, moderate, and high quality, respectively (see Tables 4 and 5).

## 3. Results

### 3.1. Study Characteristics

Eight eligible studies were conducted across five SSA countries: Kenya [29, 31], South Africa [25, 30], Zambia [26, 27], Tanzania [24], and Uganda [28]. Five studies used randomized controlled trial designs, two employed mixed methods [29, 31], and one used a quasiexperimental design [30]. The total sample size was 1510 and varied considerably from 30 in the Kenya WhatsApp study [29] to 610 in the Zambian trauma-focused cognitive behavioral therapy (TF-CBT) [26]. The participants aged between 7 and 24 years (see Table 2).

### 3.2. Mental Health Interventions Targeting YPLHIV

Eight studies reported on 8 different but interrelated interventions designed to address mental health burden in YPLHIV as well as improve adherence to ART. The interventions and their respective characteristics are summarized in Table 3. As can be seen, some interventions were delivered over 10 weeks (e.g., Sauti ya Vijana [SYV] in Tanzania) [24], while others lasted up to 6 months (e.g., WhatsApp intervention in Kenya). Follow-up periods ranged from immediate postintervention assessments [27] to 6 months [28–30]. The interventions share similarities and differences in the mode of delivery, duration, and core components/essential characteristics. Majority of the interventions used lay counselors or community members to deliver the intervention, likely addressing the shortage of mental health professionals in these settings. Following a critical review of the interventions, the interventions were regrouped under the broad categories described below.

#### 3.2.1. CBT-Based Interventions

CBT is a type of psychological treatment proven to be effective for a range of problems, including depression and anxiety disorders [32]. It is one of the evidence-based interventions for a range of behavioral and mental health problems for diverse populations and age groups, such as YPLHIV [33]. We identified four interventions that were based on the ethos and principles of CBT. The SYV (The Voice of Youth) intervention incorporated CBT along with Interpersonal Psychotherapy and Motivational Interviewing. The intervention was cocreated with the youth who were the intended beneficiaries and was delivered by trained lay group leaders over 10 weeks through in-person group sessions, including individual and joint sessions with caregivers [24]. The group-based cognitive behavioral therapy (G-CBT) was delivered over 6 months by trained health paracounselors. This intervention focused on core CBT components such as psychoeducation, cognitive restructuring, and skill-building, designed to address HIV/AIDS stigma, depression, mood management, and related issues. The G-CBT also incorporated some elements of multiple family group therapy sessions, focusing on core components such as rules, responsibility, relationships, respectful communication, stress, and social support [28]. The TF-CBT intervention with enhanced psychosocial counseling was designed to specifically address trauma and related problems. It was delivered over 12 weeks by lay counselors and includes elements such as psychoeducation, relaxation techniques, and trauma narrative work [26]. Lastly, the Problem Management Plus (PM+) is a brief, transdiagnostic psychological intervention based on CBT principles. It was initially developed by the WHO and adapted and delivered via mobile phone in Kenya in a 10-week session, facilitated by trained lay helpers. The PM+ is focused on stress management, problem-solving, and behavioral activation [31].

#### 3.2.2. Family-Based Interventions

Family-based interventions are structured therapeutic approaches that involve family members in addressing mental health challenges, focusing on improving communication, support, and collective coping strategies to enhance individual and family well-being [34]. Studies evaluating the involvement of families in intervention programming have generally reported positive impacts [35, 36]. We identified one intervention for YPLHIV that was designed along family systems and resources: The VUKA Family Intervention. Designed to improve psychosocial functioning and health outcomes, the VUKA Family Intervention was delivered over 3 months through group sessions involving (1) family group activities and (2) separate parent and child group activities. It uses a culturally tailored cartoon storyline to address various aspects of living with HIV, including disclosure, adherence, and caregiver–child communication [25].

#### 3.2.3. Peer Support Interventions

Peer-based interventions are mental health support strategies that leverage individuals with lived experience of similar mental health challenges to provide support, guidance, and mutual understanding through shared experiences [37]. Peer support played a crucial role in two interventions. The first intervention, a mobile-based mental health peer support program, utilized the WhatsApp platform to address mental health and related issues. The intervention delivery spans 6 months and it employs online groups and direct messaging techniques. This program was facilitated by a trained pediatric HIV adherence and disclosure counselor [29]. The second identified peer support intervention consists of in-person group meetings held over 10 weeks and focused on providing information about HIV/AIDS, encouraged child-initiated discussions, and conducting group activities. This intervention was delivered by moderators and a psychiatrist [27].

#### 3.2.4. Community-Based Interventions

Community-based interventions represent a powerful, holistic approach to mental health that extends beyond traditional clinical models, particularly in contexts with limited healthcare infrastructure [38]. Only one intervention fell under this category. The “Make A Difference” (MAD) intervention implemented in South Africa is a community-based approach delivered over 6 months by trained “youth ambassadors.” The MAD intervention used art and education activities as part of strategies to build self-worth, self-concept, and emotional control among HIV-positive youth [30].

### 3.3. Effectiveness of the Interventions

#### 3.3.1. Effectiveness of the Intervention on Depressive Symptoms

Depression was a common primary outcome and was assessed in 7 studies from 5 countries [24, 25, 27–31] and was measured using validated tools. Two studies in Kenya [29, 31] and one in Tanzania [24] utilized the Patient Health Questionnaire (PHQ-9); two studies in South Africa [25, 30] and one in Uganda [28] used the Child Depression Inventory (CDI) adapted for their respective contexts, while one study in Zambia used the Hamilton Depression Scale for Children [27].

The interventions showed mixed results in addressing depression among HIV-positive youth. Three interventions demonstrated significant effects on depressive symptoms [27, 28, 31]. The peer support intervention in Zambia reported a notable reduction in depression cases from 42.5% to 15% postintervention, with a significant decrease in severe depression cases from 13 to 3 [27]. The Multiple Family Group-Family Strengthening (MFG-FS) intervention in Uganda significantly reduced depressive symptoms [28], and the PM+ caused a significant reduction in mean depressive symptoms (4.9 v. 8.9; *p*=0.002) [31]. In contrast, four interventions showed no substantial change [24, 25, 29, 30]. Specifically, the SYV program in Tanzania, the VUKA Family Intervention in South Africa, the MAD community–based intervention, and the mobile-based WhatsApp intervention in Kenya showed no significant changes in depression scores between intervention and control groups [24, 25, 29, 30].

#### 3.3.2. Effectiveness of the Intervention on Anxiety Symptoms

Only one study explicitly reported on anxiety outcomes in Kenya using the validated Generalized Anxiety Disorder Scale (GAD-7), following the adaptation and implementation of the PM+ intervention [31]. At the end of the intervention, there was a sharp and significant reduction in mean anxiety symptom scores (4.6 v. 7.0; *p*=0.030) in the intervention group compared to the waitlist group. The effectiveness of the intervention was reported at follow-up [31].

#### 3.3.3. Effectiveness of the Interventions on Adherence to ART

Adherence to ART was a secondary key outcome and was measured through self-reported measures in Tanzania [24], Uganda [28], and South Africa [25]. One study in Kenya [29] used the Comprehensive ART Measure for Pediatrics (CAMP) Adherence Questionnaire and electronic dose monitoring via edical Event Monitoring System (MEMS). Four interventions demonstrated positive impacts of mental health interventions on adherence to ART [24, 25, 28, 29], although the measure and magnitude of improvement varied across studies.

The mobile-based WhatsApp intervention in Kenya reported improvement in self-reported adherence and a decrease in missed doses over 6 months. Electronic dose monitoring via MEMS showed that 59% of participants were adherent, providing objective support for the intervention's effectiveness [29]. The VUKA Family Intervention in South Africa reported improvement in ART adherence in the intervention group compared to the control group [25]. In Uganda, the G-CBT participants reported better ART adherence compared to the MFG-FS intervention at 6 months postintervention [28]. The SYV program in Tanzania also showed a slight improvement in self-reported ART adherence at 6 months, although the improvement was not statistically significant [24].

## 4. Discussion

This synthesis provides a comprehensive overview of mental health interventions for YPLHIV in SSA. Key findings of the review are discussed below.

### 4.1. Classification of Mental Health Interventions

The review identified four main categories of interventions: CBT-based, family-based, peer support, and community-based interventions. This diversity of approaches aligns with previous reviews which also found a range of intervention types for HIV-affected youth in low- and middle-income countries [17]. However, the current synthesis highlights a growing emphasis on CBT-based interventions, which were less prominent in earlier reviews [17, 18]. Four of the interventions utilized CBT principles, including adaptations such as TF-CBT and PM+. The prominence of CBT-based interventions is consistent with global trends in mental health treatment. A previous meta-analysis found CBT to be effective for various mental health conditions across different cultural contexts [16]. The variety of intervention types suggests that a one-size-fits-all approach may not be suitable for addressing mental health in HIV-positive youth [39, 40].

It is crucial to highlight that the intervention development or adaptation processes were underpinned by cocreation methodologies or demonstrated significant involvement of the intended beneficiaries of the interventions. This is important to ensure that the interventions are culturally and linguistically appropriate. Closely related to the above is the observation that most of the interventions utilized lay counselors or community members as intervention providers. This approach is consistent with the task-shifting model advocated by the WHO [41]. A review found that psychological treatments delivered by nonspecialist providers were effective in low- and middle-income countries [42]. Task shifting to lay providers is highly recommended as a strategy to address the shortage of mental health professionals in resource-limited settings. Using existing resources and structures to support the delivery of interventions is one of the surest ways to promote the sustainability of interventions beyond the funding period.

### 4.2. Effectiveness of Interventions Against Selected Outcomes

Depression was the most assessed outcome, followed by ART adherence. Anxiety was less measured. Most studies used widely recognized and validated tools to assess the impact of the interventions on the chosen outcomes. This strengthens as well as enhances the reliability and validity of the findings [43], allowing for more confidence in the results and potential comparability across studies. The increasing focus on depression aligns with previous research highlighting its prevalence among HIV-positive adolescents and youth [6, 9]. However, the limited assessment of anxiety contrasts with studies suggesting high comorbidity of anxiety and depression in this population [44, 45]. The inclusion of ART adherence as a secondary outcome in this review and several studies included in this review reflects growing recognition of the bidirectional relationship between mental health and HIV treatment adherence, a link that has been increasingly emphasized in recent years [46, 47].

The findings present a mixed picture of effectiveness for mental health interventions among HIV-positive youth in Africa. The peer support intervention in Zambia showed significant reductions in depressive symptoms [27]. This aligns with previous research suggesting that family-based and peer support interventions can be effective in improving mental health outcomes for YPLHIV [17, 48, 49]. In contrast, the SYV in Tanzania and the VUKA Family Intervention in South Africa did not show significant improvements in depression scores. This variability in outcomes suggests that the effectiveness of mental health interventions may be context-dependent and influenced by factors such as cultural appropriateness, delivery method, and intensity of the intervention [50–52]. As mentioned earlier, the mixed results imply that a one-size-fits-all approach to mental health interventions for HIV-positive youth may not be effective [40]. There is, therefore, a need for more research to understand which components of successful interventions contribute most to positive outcomes. Cultural adaptation of interventions may be crucial for their effectiveness in different African contexts.

The limited data on anxiety interventions make it difficult to draw broader conclusions on the effectiveness of some interventions. However, the positive results from the PM+ intervention are encouraging. The effectiveness of PM+ aligns with findings from other contexts, such as those reported by previous researchers in their study of PM+ for adults with mental health disorders [53, 54]. The findings of this review indicate that there is a considerable lack of research on anxiety interventions for HIV-positive youth in Africa, making it ethically and scientifically valid to highlight this gap. Nonetheless, mobile-delivered interventions may show potential for addressing anxiety.

Several interventions demonstrated positive effects on ART adherence. The mobile-based WhatsApp intervention in Kenya, the VUKA Family Intervention in South Africa, and the G-CBT in Uganda all improved adherence to ART. The generally positive effects on ART adherence across different intervention types are encouraging. These findings align with previous reviews [55, 56] which found that various types of interventions could improve adherence among HIV-positive youth. The link between mental health improvement and better ART adherence suggests that addressing mental health could have broader positive impacts on HIV management [57].

### 4.3. Implications for Practice and Policy

Practitioners should implement integrated care models addressing both mental health and HIV management, prioritizing peer support and family strengthening programs for their effectiveness in reducing depression. Multicomponent interventions that target mental health and ART adherence should be implemented and assessed using objective measures. Policymakers should support frameworks for mobile and digital technologies in mental health interventions due to their scalability and effectiveness, especially in areas with limited access to in-person services. Mental health professionals and peer supporters must be trained in culturally competent care, and comprehensive mental health assessments, including measures of depression, and anxiety should be part of routine care for HIV-positive youth.

### 4.4. Implications for Future Research and Interventions

Research should focus on developing standardized outcome measures for comparison and meta-analysis, with more longitudinal studies to assess the long-term impacts on mental health and ART adherence. Limited longitudinal evidence exists for mental health interventions targeting YPLHIV in SSA. Most existing studies provide cross-sectional insights rather than long-term outcome tracking. Increased research on anxiety interventions for HIV-positive youth is needed to fill current knowledge gaps. Identifying effective components of interventions can aid in developing potent multicomponent programs, and cost-effectiveness analyses should inform policy and implementation decisions. Future research should focus on comparing the effectiveness of different intervention types within the same cultural context to identify the most appropriate approaches. Researchers should also investigate cultural adaptation of interventions for different African contexts and explore technology-based interventions' acceptability, feasibility, and effectiveness. Qualitative research is essential to understand HIV-positive youths' experiences with mental health interventions. Implementation science research is needed to scale successful interventions in real-world settings, and studies should examine combined evidence-based approaches. Investigating protective factors and resilience among HIV-positive youth can inform strength-based interventions. Standardized training programs for lay providers should be developed and evaluated with ongoing supervision and support systems to ensure quality of service provision by lay personnel. Similarly, it was observed that validated tools were used to assess the review outcomes. However, the variability observed in the assessment tool type and item number can make direct comparisons between studies more challenging. Standardizing the use of specific tools across similar studies in the region [58] may facilitate easier comparisons and potential meta-analyses. The most frequently used tool for assessing treatment adherence was self-reported measures. Although self-reports can offer valuable insights into patient experiences, they are susceptible to recall and social desirability biases, which might lead to inaccurate estimates of adherence rates [59–61]. Future research should aim to balance validated, culturally adapted tools with more objective measures for adherence.

### 4.5. Limitations of the Review

This systematic review has several limitations that affect the interpretation of its findings and future research planning. The small number of studies meeting the inclusion criteria limits generalizability and may not fully represent the diverse range of interventions and contexts across Africa. Geographical representation is also limited, as the studies were conducted in only a few African countries, overlooking regional variations. The variability in outcome measures and the use of self-reported data complicate the synthesis and comparison of results. Many studies had short follow-up periods, leaving the long-term effectiveness of interventions unknown. The current review's quantitative focus represents a potential limitation, recognizing that nuanced experiences and contextual insights may not be fully captured by purely quantitative methods. Additionally, there is a scarcity of studies addressing anxiety and overall mental health functioning, limiting our understanding of comprehensive mental health interventions for HIV-positive youth in Africa. The imposition of date restrictions and limiting the review to English-language publications may have excluded potentially relevant studies and overlooked valuable non-English research, limiting the scope of inclusivity.

## 5. Conclusions

This systematic review reveals a complex landscape of mental health interventions for HIV-positive youth in Africa. While some interventions show promise in addressing depression and improving ART adherence, results are mixed and often context-dependent. Peer support, family-based interventions, and mobile health approaches emerge as potentially effective strategies. However, significant gaps remain, particularly in addressing anxiety and overall mental health functioning. The review underscores the need for more rigorous, standardized, and culturally adapted research across diverse African settings. Future efforts should focus on developing integrated, multicomponent interventions that address both mental health and HIV management, with an emphasis on long-term effectiveness and scalability. Ultimately, this review highlights the critical importance of mental health support in HIV care for youth in Africa and calls for increased attention and resources in this vital area of public health.

## Figures and Tables

**Table 1:** Search strategy.

Electronic database	Search strategy
PubMed	Adolescent [MeSH Terms] OR Adolescen^∗^[Text Word] OR “young adult^∗^” [Text Word] OR teen^∗^[Text Word] OR youth [Text Word] “young people” [Text Word] OR “young women” [Text Word] OR “young men” [Text Word] OR “young person” [Text Word] OR “10–19 year olds” [Text Word] AND hiv [MeSH Terms] OR HIV [Text Word] OR “human immunodeficiency virus” [Text Word] OR AIDS [Text Word] OR “HIV/AIDS” [Text Word] OR “HIV and AIDS” [Text Word] OR “acquired immunodeficiency syndrome” [Text Word] OR “immune failure” [Text Word] OR “HIV-positive” [Text Word] OR SADA [Text Word] AND “Mental health intervention” [text Word] OR “school-based intervention” [Text Word] OR “community-based intervention” [text Word] OR “peer intervention” [text Word] OR “church-based intervention” [text Word] OR “family-based intervention” [Text Word] OR “group based intervention” [Text Word] OR “cognitive behavior therap^∗^” [Text Word]OR “psychosocial intervention” [MeSH Terms] OR Psychosocial intervention^∗^[text Word] OR “Psychological intervention” [Text Word] OR “interpersonal therap^∗^” [text Word] OR “group therapy” [Text Word] OR “behavior therap^∗^” [text Word]OR “cognitive therap^∗^” [text Word] AND Africa [text Word] OR “Sub Saharan Africa” [Text Word]
MEDLINE	TX (Adolescen^∗^ OR “young adult^∗^”OR teen^∗^ OR youth OR “young people” OR “young women” OR “young men” OR “young person” OR “10–19 year olds”) AND TX (HIV OR “human immunodeficiency virus” OR AIDS OR “HIV/AIDS” OR “HIV and AIDS”OR “acquired immunodeficiency syndrome” OR “immune failure” OR “HIV-positive” OR SADA) AND TX (“Mental health intervention” OR “school-based intervention” OR “community-based intervention” OR “peer intervention” OR “church-based intervention” OR “family-based intervention^∗^” OR “health facility-based intervention” OR “group based interventions” OR “cognitive behavioural therap^∗^” OR “Psychosocial intervention” OR “Psychological intervention” OR “interpersonal therap^∗^” OR “group therap^∗^” OR “behaviour therap^∗^” OR “Cognitive therap^∗^”) AND TX (Africa OR “Sub Saharan Africa”)
CINAHL	TX (Adolescen^∗^ OR “young adult^∗^”OR teen^∗^ OR youth OR “young people” OR “young women” OR “young men” OR “young person” OR “10–19 year olds”) AND TX (HIV OR “human immunodeficiency virus” OR AIDS OR “HIV/AIDS” OR “HIV and AIDS”OR “acquired immunodeficiency syndrome” OR “immune failure” OR “HIV-positive” OR SADA) AND TX (“Mental health intervention” OR “school-based intervention” OR “community-based intervention” OR “peer intervention” OR “church-based intervention” OR “family-based intervention^∗^” OR “health facility-based intervention” OR “group based interventions” OR “cognitive behavioural therap^∗^” OR “Psychosocial intervention” OR “Psychological intervention” OR “interpersonal therap^∗^” OR “group therap^∗^” OR “behaviour therap^∗^” OR “Cognitive therap^∗^”) AND TX (Africa OR “Sub Saharan Africa”)
Scopus	TX (Adolescen^∗^ OR “young adult^∗^”OR teen^∗^ OR youth OR “young people” OR “young women” OR “young men” OR “young person” OR “10–19 year olds”) AND TX (HIV OR “human immunodeficiency virus” OR AIDS OR “HIV/AIDS” OR “HIV and AIDS” OR “acquired immunodeficiency syndrome” OR “immune failure” OR “HIV-positive” OR SADA) AND TX (“Mental health intervention” OR “school-based intervention” OR “community-based intervention” OR “peer intervention” OR “church-based intervention” OR “family-based intervention^∗^” OR “health facility-based intervention” OR “group based interventions” OR “cognitive behavioural therap^∗^” OR “Psychosocial intervention” OR “Psychological intervention” OR “interpersonal therap^∗^” OR “group therap^∗^” OR “behaviour therap^∗^” OR “Cognitive therap^∗^”) AND TX (Africa OR “Sub Saharan Africa”)
Web of Science	TX (Adolescen^∗^ OR “young adult^∗^”OR teen^∗^ OR youth OR “young people” OR “young women” OR “young men” OR “young person” OR “10–19 year olds”) AND TX (HIV OR “human immunodeficiency virus” OR AIDS OR “HIV/AIDS” OR “HIV and AIDS” OR “acquired immunodeficiency syndrome” OR “immune failure” OR “HIV-positive” OR SADA) AND TX (“Mental health intervention” OR “school-based intervention” OR “community-based intervention” OR “peer intervention” OR “church-based intervention” OR “family-based intervention^∗^” OR “health facility-based intervention” OR “group based interventions” OR “cognitive behavioural therap^∗^” OR “Psychosocial intervention” OR “Psychological intervention” OR “interpersonal therap^∗^” OR “group therap^∗^” OR “behaviour therap^∗^” OR “Cognitive therap^∗^”) AND TX (Africa OR “Sub Saharan Africa”)

**Table 2:** Summary of data extracted from included studies.

Author/year	Country	Study design	Participant characteristics	Intervention type	Study outcomes & their measures	Effectiveness of intervention
Dow et al., 2022	Tanzania	Pilot randomized controlled trial	130 young people living with HIV (YPLWH) aged 12–24 years.	Sauti ya Vijana (SYV; The Voice of Youth)	Depression; Patient Health Questionnaire (PHQ-9), ART adherence; three-question self-reported survey.	No significant improvements in mental health (depression); slight improvement in self-reported ART adherence at 6 months.

Chory et al., 2021	Kenya	Prospective mixed method study	30 adolescents living with HIV (ALWH) aged 10–19 years	Mobile-based mental health and peer support intervention using WhatsApp® platform	Adherence to ART; Comprehensive ART Measure for Pediatrics (CAMP) Adherence Questionnaire and electronic dose monitoring via Medical Event Monitoring System (MEMS) Depression; Patient Health Questionnaire-9 (PHQ-9) and Hopkins symptom checklist.	Improvement in self-reported adherence, decrease in missed doses over 6 months, MEMS data showed 59% of participants were adherent. There was no discernible change over time in respondents' depression scores

Bhana et al., 2014	South Africa	Randomized controlled trial	65 adolescents aged 10–14 years enrolled in HIV care and aware of their HIV status	The VUKA Family Intervention	Depression; Child Depression Inventory (CDI), adherence to ART; self-reports	Improved ART adherence in the VUKA group compared to the control group, no significant changes in depression score among the intervention and control group.

Nyongesa et al., 2022	Kenya	Mixed-method formative research (qualitative and quantitative methods)	70 young persons living with HIV (aged 18–24 years) with mild-to-moderate symptoms of common mental disorders (CMDs)	Problem Management Plus (PM+), adapted for mobile phone delivery	Depression; Patient Health Questionnaire (PHQ-9). Anxiety; Generalised Anxiety Disorder Scale (GAD-7).	At endline, a sharp and significant reduction in mean depressive (4.9 v. 8.9; *p*=0.002) and anxiety symptom (4.6 v. 7.0; *p*=0.030) scores was observed in the intervention compared with the waitlist group and effect sustained at follow-up

Mueller et al., 2010	South Africa	Quasiexperimental, cross-sectional postintervention design	297 children and adolescents aged 8–18 years diagnosed with HIV	Community-based intervention called “Make A Difference” (MAD)	Depression; Measured using an adapted version of the Child Depression Inventory (CDI)	No statistically significant differences were found between those who attended the intervention and those who did not on the CDI (depression) and the SDQ (emotional and behavioral problems)

Menon et al., 2014	Zambia	Randomized controlled trial (RCT)	130 children recruited from three antiretroviral (ART) clinics	Peer support intervention	Depression; Hamilton Depression Scale for children.	Children with depression reduced from 42.5% to 15% postintervention. There was significant reduction in severity: Severe depression cases reduced from 13 to 3.

Kane et al., 2023	Zambia	Randomized controlled trial	610 orphaned adolescents aged 13–17 years old.	Trauma-Focused Cognitive Behavioral Therapy (TF-CBT) with Enhanced Psychosocial Counseling (PC+)	Mental health functioning; 61 item self-reported survey	No significant difference between group treatment effects for HIV risk behaviors and mental health outcomes

Nabunya et al., 2024	Uganda	Cluster randomized controlled trial	Adolescents living with HIV (10–14 years) and their caregivers. 89 adolescent-caregiver dyads (*n* = 178)	Group-based Cognitive Behavioral Therapy (G-CBT) Multiple Family Group-Family Strengthening (MFG-FS)	Depression; 14-item Child Depression Inventory, adherence to ART; Wilson's three-item self-reported adherence measure	MFG-FS significantly reduced depressive symptoms at 3 months. G-CBT participants had better ART adherence compared to MFG-FS at 6 months.

**Table 3:** Characteristics of the intervention.

Author/year	Intervention type	Duration of intervention	Mode of delivery	Providers of the intervention	Components of the intervention
Dow et al., 2022	Sauti ya Vijana (SYV; The Voice of Youth)	10 weeks	In-person; group sessions, including individual and joint sessions with caregivers.	Trained Tanzanian group leaders	Based on Cognitive Behavioral Therapy, Interpersonal Psychotherapy, and Motivational Interviewing.

Chory et al., 2021	Mobile-based mental health and peer support intervention using WhatsApp® platform.	6 months	Online via WhatsApp® groups, direct messaging via WhatsApp®	Trained pediatric HIV adherence and disclosure counselor	A smartphone with the WhatsApp® application preinstalled, a SIM card, and phone credit (∼7 USD per month). WhatsApp® groups for 9–14 and 15–19 year-olds, weekly group discussion topics, biweekly individual check-ins via WhatsApp

Bhana et al., 2014	The VUKA Family Intervention	3 months	Group sessions involving both multiple family group activities and separate parent and child group activities	Lay counselors	A culturally-tailored cartoon storyline and curriculum focused on (1) AIDS-related loss and bereavement; (2) HIV transmission and treatment knowledge; (3) disclosure of HIV status to others; (4) youth identity, acceptance, and coping with HIV; (5) adherence to medical treatment; (6) stigma and discrimination; (7) caregiver–child communication, particularly on sensitive topics such as puberty and HIV; (8) puberty; (9) identifying and developing strategies to keep children safe in high-risk situations where sexual behavior and drug use are possible; and (10) social support

Nyongesa et al., 2022	Problem Management Plus (PM+), adapted for mobile phone delivery	Ten weekly sessions	Online via mobile phone	Trained lay helpers recruited from the community.	Stress management, problem-solving, behavioral activation, social support, and psychoeducation about common reactions to adversity.

Mueller et al., 2010	Community-based intervention called “Make A Difference” (MAD)	6 months (50+ sessions)	In person, group based.	Trained ‘youth ambassadors'	Method: art and education activities Activities: creating “hero” books about personal life journeys, group HIV education activities focused on self-advocacy and empowerment Goal: build self-worth (self-esteem), self-concept, empowerment, and emotional control (self-efficacy)

Menon et al., 2014	Peer support intervention	10 weeks	In person group meetings	Moderators and a psychiatrist	Materials: Brochure developed by the Department of Psychology at the University of Zambia. Methods: • Reviewing group rules • Imparting information on HIV and AIDS • Participating in child-initiated talk time • Group activities • Free play

Kane et al., 2023	Trauma-Focused Cognitive Behavioral Therapy (TF-CBT) with Enhanced Psychosocial Counseling (PC+)	12 weeks	In person, group based.	Lay counselors with minimal previous mental health or counseling experience	Psychoeducation, parenting skills, relaxation, affective modulation, cognitive coping, trauma narrative, in vivo exposure, conjoint session, and enhancing safety skills.

Nabunya et al., 2024	Group-based Cognitive Behavioral Therapy (G-CBT) Multiple Family Group-Family Strengthening (MFG-FS	6 months	In-person group sessions	G-CBT: trained health paracounselors. MFG-FS: trained parent peers	Core components of CBT: psychoeducation, cognitive restructuring, and skill-building. Topics: HIV/AIDS stigma and depression, thoughts and emotions, challenging negative thoughts, setting goals, visualization, mood management, etc. Core components of MFG. Topics: HIV/AIDS knowledge and adherence, stigma, building family support, problem-solving, dealing with stress, family relationships

**Table 4:** Methodological quality and risk of bias assessment for randomized controlled trials.

Study	Sequence generation		Blinding of participants and outcome assessors	Incomplete outcome data	Selective outcome reporting	Other sources of potential bias	Overall quality of study
	Was the allocation sequence adequately generated?	Was allocation adequately concealed?	Was knowledge of the allocated intervention adequately prevented during the study?	Were incomplete outcome data adequately addressed?	Are reports of the study free of suggestion of selective outcome reporting?	Was the study free of other problems that could put it at a high risk of bias?	
[24]	Yes	Yes	Yes	Yes	Yes	No	5; high quality
[25]	Yes	Unclear	Yes	No	Yes	No	3; moderate quality
[26]	Yes	No	No	Yes	Yes	No	3; moderate quality
[27]	Unclear	unclear	Unclear	Yes	Yes	Yes	3; moderate quality
[28]	Yes	Yes	Yes	Yes	Yes	Yes	6; high quality

**Table 5:** Methodological quality and risk of bias assessment for nonrandomized controlled studies.

Study	Participant selection		Exposure to intervention		Comparability	Assessment of outcomes				Other potential sources of bias	Overall quality of study
	Were participants selected to be representative of the target population?	Were there clear participant selection criteria (for both exposed and control group) avoiding inappropriate exclusions?	Was a comparison/control group assessed?	Was the intervention being studied, applied consistently to all eligible participants?	Were potential confounders identified and appropriately adjusted for?	Were procedures for assessment of outcome sufficient to satisfy confirmation of presence of condition of interest	Was follow-up long enough for outcome to occur?	Were incomplete outcome data adequately addressed?	Are reports of the study free of suggestion of selective outcome reporting?	Was the study free of other problems that could put it at a high risk of bias?	
[29]	No	Yes	Yes	Yes	Unclear	Yes	Yes	Unclear	Yes	Yes	7; high quality
[30]	Yes	Yes	Yes	Yes	No	Yes	Yes	No	Yes	No	7; high quality
[31]	Yes	Yes	Yes	Yes	No	Yes	Unclear	Unclear	Yes	No	6; moderate quality

## Data Availability

The data that support the findings of this study are available on request from the corresponding author. The data are not publicly available due to privacy or ethical restrictions.

## Supporting Information

PRISMA checklist.

## References

[1] Anokye R., Acheampong E., Budu-Ainooson A., Obeng E. I., Akwasi A. G. (2018). Prevalence of Postpartum Depression and Interventions Utilized for its Management. *Annals of General Psychiatry*.

[2] UNAIDS (2022). Fact Sheet: Latest Global and Regional Statistics on the Status of the AIDS Epidemic. https://www.unaids.org/en/resources/documents/2022/UNAIDS_FactSheet.

[3] Kharsany A. B., Karim Q. A. (2016). HIV Infection and AIDS in Sub-saharan Africa: Current Status, Challenges and Opportunities. *The Open AIDS Journal*.

[4] Wright N., Hill J., Sharp H., Pickles A. (2021). Interplay between Long‐term Vulnerability and New Risk: Young Adolescent and Maternal Mental Health Immediately before and during the COVID‐19 Pandemic. *JCPP advances*.

[5] Dessauvagie A. S., Jörns-Presentati A., Napp A.-K. (2020). The Prevalence of Mental Health Problems in Sub-saharan Adolescents Living with HIV: a Systematic Review. *Global Mental Health*.

[6] Ayano G., Demelash S., Abraha M., Tsegay L. (2021). The Prevalence of Depression Among Adolescent with HIV/AIDS: a Systematic Review and Meta-Analysis. *AIDS Research and Therapy*.

[7] Woollett N., Cluver L., Bandeira M., Brahmbhatt H. (2017). Identifying Risks for Mental Health Problems in HIV Positive Adolescents Accessing HIV Treatment in Johannesburg. *Journal of Child and Adolescent Mental Health*.

[8] Brooks M., Burmen B., Olashore A. (2023). Symptoms of Depression, Anxiety, and Thoughts of Suicide/self-Injury in Adolescents and Young Adults Living with HIV in Botswana. *African Journal of AIDS Research*.

[9] Abebe H., Shumet S., Nassir Z., Agidew M., Abebaw D. (2019). Prevalence of Depressive Symptoms and Associated Factors Among HIV‐positive Youth Attending ART Follow‐up in Addis Ababa, Ethiopia. *AIDS research and treatment*.

[10] Musisi S., Kinyanda E. (2009). Emotional and Behavioural Disorders in HIV Seropositive Adolescents in Urban Uganda. *East African Medical Journal*.

[11] Remien R. H., Stirratt M. J., Nguyen N., Robbins R. N., Pala A. N., Mellins C. A. (2019). Mental Health and HIV/AIDS: the Need for an Integrated Response. *AIDS*.

[12] Dvalishvili D., Ssewamala F. M., Nabunya P. (2022). Impact of Family-Based Economic Empowerment Intervention, Suubi+ Adherence (2012–2018) on Multidimensional Poverty for Adolescents Living with HIV (ALWHIV) in Uganda. *International Journal of Environmental Research and Public Health*.

[13] Novella E. J. (2010). Mental Health Care in the Aftermath of Deinstitutionalization: a Retrospective and Prospective View. *Health Care Analysis*.

[14] World Health Organization, Association W. P., Child I. A. f., Psychiatry A., Professions A. (2005). *Atlas: Child and Adolescent Mental Health Resources: Global Concerns, Implications for the Future*.

[15] Nakimuli-Mpungu E., Musisi S., Smith C. M. (2021). Mental Health Interventions for Persons Living with HIV in Low‐and Middle‐income Countries: a Systematic Review. *Journal of the International AIDS Society*.

[16] Asrat B., Schneider M., Ambaw F., Lund C. (2020). Effectiveness of Psychological Treatments for Depressive Symptoms Among People Living with HIV/AIDS in Low-And Middle-Income Countries: A Systematic Review and Meta-Analysis. *Journal of Affective Disorders*.

[17] Bhana A., Abas M. A., Kelly J., Van Pinxteren M., Mudekunye L. A., Pantelic M. (2020). Mental Health Interventions for Adolescents Living with HIV or Affected by HIV in Low-And Middle-Income Countries: Systematic Review. *BJPsych open*.

[18] Bhana A., Kreniske P., Pather A., Abas M. A., Mellins C. A. (2021). Interventions to Address the Mental Health of Adolescents and Young Adults Living with or Affected by HIV: State of the Evidence. *Journal of the International AIDS Society*.

[19] Mabrouk A., Mbithi G., Chongwo E. (2022). Mental Health Interventions for Adolescents in Sub-saharan Africa: A Scoping Review. *Frontiers in Psychiatry*.

[20] Page M. J., McKenzie J. E., Bossuyt P. M. (2021). The PRISMA 2020 Statement: an Updated Guideline for Reporting Systematic Reviews. *BMJ*.

[21] Schreier M. (2012). Qualitative Content Analysis in Practice.

[22] Higgins J. P., Altman D. G., Gotzsche P. C. (2011). The Cochrane Collaboration’s Tool for Assessing Risk of Bias in Randomised Trials. *BMJ*.

[23] Wells G. A. S., O’Connell B., Peterson D., Welch V., Losos M., Tugwell P. (2021). The Newcastle-Ottawa Scale (NOS) for Assessing the Quality of Nonrandomised Studies in Meta-Analyses. https://www.ohri.ca/programs/clinical_epidemiology/oxford.asp.

[24] Dow D. E., O’Donnell K. E., Mkumba L. (2022). Sauti Ya Vijana (SYV; the Voice of Youth): Longitudinal Outcomes of an Individually Randomized Group Treatment Pilot Trial for Young People Living with HIV in Tanzania. *AIDS and Behavior*.

[25] Bhana A., Mellins C. A., Petersen I. (2014). The VUKA Family Program: Piloting a Family-Based Psychosocial Intervention to Promote Health and Mental Health Among HIV Infected Early Adolescents in South Africa. *AIDS Care*.

[26] Kane J. C., Figge C., Paniagua-Avila A. (2024). Effectiveness of Trauma-Focused Cognitive Behavioral Therapy Compared to Psychosocial Counseling in Reducing HIV Risk Behaviors, Substance Use, and Mental Health Problems Among Orphans and Vulnerable Children in Zambia: a Community-Based Randomized Controlled Trial. *AIDS and Behavior*.

[27] Menon J., Paul R., Nkumbula T., Lwatula C., Musepa M., Ngoma M. (2014). Impact of HIV Information and Peer Support on Psychiatric Outcomes in HIV Positive Young People. *Medical Journal of Zambia*.

[28] Nabunya P., Ssewamala F. M., Kizito S. (2024). Preliminary Impact of Group-Based Interventions on Stigma, Mental Health, and Treatment Adherence Among Adolescents Living with Human Immunodeficiency Virus in Uganda. *The Journal of Pediatrics*.

[29] Chory A., Callen G., Nyandiko W. (2022). A Pilot Study of a Mobile Intervention to Support Mental Health and Adherence Among Adolescents Living with HIV in Western Kenya. *AIDS and Behavior*.

[30] Mueller J., Alie C., Jonas B., Brown E., Sherr L. (2011). A Quasi‐experimental Evaluation of a Community‐based Art Therapy Intervention Exploring the Psychosocial Health of Children Affected by HIV in South Africa. *Tropical Medicine and International Health*.

[31] Nyongesa M. K., Mwangome E., Mwangi P. (2022). Adaptation, Acceptability and Feasibility of Problem Management Plus (PM+) Intervention to Promote the Mental Health of Young People Living with HIV in Kenya: Formative Mixed-Methods Research. *BJPsych open*.

[32] Hofmann S. G., Asnaani A., Vonk I. J., Sawyer A. T., Fang A. (2012). The Efficacy of Cognitive Behavioral Therapy: A Review of Meta-Analyses. *Cognitive Therapy and Research*.

[33] Wenzel A. (2017). Basic Strategies of Cognitive Behavioral Therapy. *Psychiatric Clinics of North America*.

[34] Healy E. A., Kaiser B. N., Puffer E. S. (2018). Family-based Youth Mental Health Interventions Delivered by Nonspecialist Providers in Low-And Middle-Income Countries: A Systematic Review. *Families, Systems & Health*.

[35] Poole L. A., Knight T., Toumbourou J. W., Lubman D. I., Bertino M. D., Lewis A. J. (2018). A Randomized Controlled Trial of the Impact of a Family-Based Adolescent Depression Intervention on Both Youth and Parent Mental Health Outcomes. *Journal of Abnormal Child Psychology*.

[36] Rodolico A., Bighelli I., Avanzato C. (2022). Family Interventions for Relapse Prevention in Schizophrenia: A Systematic Review and Network Meta-Analysis. *The Lancet Psychiatry*.

[37] Lyons N., Cooper C., Lloyd-Evans B. (2021). A Systematic Review and Meta-Analysis of Group Peer Support Interventions for People Experiencing Mental Health Conditions. *BMC Psychiatry*.

[38] Castillo E. G., Ijadi-Maghsoodi R., Shadravan S. (2019). Community Interventions to Promote Mental Health and Social Equity. *Current Psychiatry Reports*.

[39] Fraser C., Judd F., Jackson H., Murray G., Humphreys J., Hodgins G. A. (2002). Does One Size Really Fit All? Why the Mental Health of Rural Australians Requires Further Research. *Australian Journal of Rural Health*.

[40] Griffiths S. L., Lalousis P. A., Wood S. J., Upthegrove R. (2022). Heterogeneity in Treatment Outcomes and Incomplete Recovery in First Episode Psychosis: Does One Size Fit All?. *Translational Psychiatry*.

[41] Hoeft T. J., Fortney J. C., Patel V., Unützer J. (2018). Task‐sharing Approaches to Improve Mental Health Care in Rural and Other Low‐resource Settings: a Systematic Review. *The Journal of Rural Health*.

[42] Ó hAnrachtaigh É., Brown G., Beck A., Conway R., Jones H., Angelakis I. (2024). Transdiagnostic Psychological Interventions for Symptoms of Common Mental Disorders Delivered by Non‐Specialist Providers in Low‐and Middle‐Income Countries: A Systematic Review and Meta‐Analysis. *Depression and Anxiety*.

[43] Tsai A. C., Scott J. A., Hung K. J. (2013). Reliability and Validity of Instruments for Assessing Perinatal Depression in African Settings: Systematic Review and Meta-Analysis. *PLoS One*.

[44] Chantaratin S., Trimetha K., Werarak P. (2022). Depression and Anxiety in Youth and Young Adults Living with HIV: Frequency and Associated Factors in Thai Setting. *Journal of the International Association of Physicians in AIDS Care*.

[45] Le Prevost M., Arenas-Pinto A., Melvin D. (2018). Anxiety and Depression Symptoms in Young People with Perinatally Acquired HIV and HIV Affected Young People in England. *AIDS Care*.

[46] Bucek A., Leu C.-S., Benson S. (2018). Psychiatric Disorders, Antiretroviral Medication Adherence and Viremia in a Cohort of Perinatally HIV-Infected Adolescents and Young Adults. *The Pediatric Infectious Disease Journal*.

[47] Carrico A. W., Rubin L. H., Paul R. H. (2022). The Interaction of HIV with Mental Health in the Modern Antiretroviral Therapy Era. *Psychosomatic Medicine*.

[48] Ahmed C. V., Doyle R., Gallagher D. (2023). A Systematic Review of Peer Support Interventions for Adolescents Living with HIV in Sub-saharan Africa. *AIDS Patient Care and STDs*.

[49] Cavazos-Rehg P., Byansi W., Xu C. (2021). The Impact of a Family-Based Economic Intervention on the Mental Health of HIV-Infected Adolescents in Uganda: Results from Suubi+ Adherence. *Journal of Adolescent Health*.

[50] Dévieux J. G., Malow R. M., Rosenberg R. (2005). Cultural Adaptation in Translational Research: Field Experiences. *Journal of Urban Health: Bulletin of the New York Academy of Medicine*.

[51] Mark D., Bloch K., Cluver L. (2018). The Power of Peers: Multi-Country Analysis of Adolescent Viral Suppression in Sub-saharan Africa. *International AIDS Conferenc*.

[52] Webb C. A., Hirshberg M. J., Davidson R. J., Goldberg S. B. (2022). Personalized Prediction of Response to Smartphone-Delivered Meditation Training: Randomized Controlled Trial. *Journal of Medical Internet Research*.

[53] Dawson K. S., Bryant R. A., Harper M. (2015). Problem Management Plus (PM+): a WHO Transdiagnostic Psychological Intervention for Common Mental Health Problems. *World Psychiatry*.

[54] Sijbrandij M., Bryant R. A., Schafer A. (2016). Problem Management Plus (PM+) in the Treatment of Common Mental Disorders in Women Affected by Gender-Based Violence and Urban Adversity in Kenya; Study Protocol for a Randomized Controlled Trial. *International Journal of Mental Health Systems*.

[55] Casale M., Carlqvist A., Cluver L. (2019). Recent Interventions to Improve Retention in HIV Care and Adherence to Antiretroviral Treatment Among Adolescents and Youth: a Systematic Review. *AIDS Patient Care and STDs*.

[56] Reif L. K., Abrams E. J., Arpadi S. (2020). Interventions to Improve Antiretroviral Therapy Adherence Among Adolescents and Youth in Low-And Middle-Income Countries: a Systematic Review 2015–2019. *AIDS and Behavior*.

[57] Boakye D. S., Setordzi M., Dzansi G., Adjorlolo S. (2024). Mental Health Burden Among Females Living with HIV and AIDS in Sub-saharan Africa: A Systematic Review. *PLOS global public health*.

[58] Obbarius A., van Maasakkers L., Baer L. (2017). Standardization of Health Outcomes Assessment for Depression and Anxiety: Recommendations from the ICHOM Depression and Anxiety Working Group. *Quality of Life Research*.

[59] Kizito S., Namuwonge F., Brathwaite R. (2022). Monitoring Adherence to Antiretroviral Therapy Among Adolescents in Southern Uganda: Comparing Wisepill to Self‐report in Predicting Viral Suppression in a Cluster‐randomized Trial. *Journal of the International AIDS Society*.

[60] Lam W. Y., Fresco P. (2015). Medication Adherence Measures: An Overview. *BioMed Research International*.

[61] Orrell C., Cohen K., Leisegang R., Bangsberg D. R., Wood R., Maartens G. (2017). Comparison of Six Methods to Estimate Adherence in an ART-Naïve Cohort in a Resource-Poor Setting: Which Best Predicts Virological and Resistance Outcomes?. *AIDS Research and Therapy*.

